# Overview and Development of the Child Health and Mortality Prevention Surveillance Determination of Cause of Death (DeCoDe) Process and DeCoDe Diagnosis Standards

**DOI:** 10.1093/cid/ciz572

**Published:** 2019-10-09

**Authors:** Dianna M Blau, J Patrick Caneer, Rebecca P Philipsborn, Shabir A Madhi, Quique Bassat, Rosauro Varo, Inácio Mandomando, Kitiezo Aggrey Igunza, Karen L Kotloff, Milagritos D Tapia, Siobhan Johnstone, Richard Chawana, Afruna Rahman, Shams El Arifeen, Dickens Onyango, Reinhard Kaiser, Anna C Seale, Nega Assefa, Timothy Morris, Pratima L Raghunathan, Robert F Breiman

**Affiliations:** 1 Center for Global Health, Centers for Disease Control and Prevention, Atlanta, Georgia, USA; 2 Public Health Informatics Institute, The Task Force for Global Health, Atlanta, Georgia, USA; 3 Emory Global Health Institute, Emory University, Atlanta, Georgia, USA; 4 Medical Research Council, Respiratory and Meningeal Pathogens Research Unit, University of the Witwatersrand, Faculty of Health Sciences, Johannesburg, South Africa; 5 Department of Science and Technology/National Research Foundation, Vaccine Preventable Diseases, University of the Witwatersrand, Faculty of Health Sciences, Johannesburg, South Africa; 6 ISGlobal, Hospital Clínic, Universitat de Barcelona, Barcelona, Spain; 7 Centro de Investigação em Saúde de Manhiça (CISM), Maputo, Mozambique; 8 Catalan Institution for Research and Advanced Studies (ICREA), Barcelona, Spain; 9 Pediatric Infectious Diseases Unit, Pediatrics Department, Hospital Sant Joan de Déu, University of Barcelona, Barcelona, Spain; 10 Consorcio de Investigacion Biomedica en Red de Epidemiologia y Salud, Spain; 11 Instituto Nacional de Saude, Ministerio de Saude, Maputo, Mozambique; 12 Kenya Medical Research Unit, Kenya Medical Research Institute (KEMRI), Kisusmu, Kenya; 13 Department of Pediatrics, Center for Vaccine Development and Global Health, University of Maryland School of Medicine, Baltimore, Maryland, USA; 14 PEI, Infectious Disease Division, icddr,b, Dhaka, Bangladesh; 15 Maternal and Child Health Division, icddr,b, Dhaka, Bangladesh; 16 Kisumu County Department of Health, Kisumu, Kenya; 17 US Centers for Disease Control and Prevention--Sierra Leone, Freetown, Sierra Leone; 18 Department of Infectious Disease Epidemiology, London School of Hygiene and Tropical Medicine, London, United Kingdom; 19 College of Health and Medical Sciences, Haramaya University, Harar, Ethiopia; 20 KEMRI–Wellcome Trust Research Programme, Kilifi, Kenya

**Keywords:** mortality review, cause of death, vital records, CHAMPS, data management

## Abstract

Mortality surveillance and cause of death data are instrumental in improving health, identifying diseases and conditions that cause a high burden of preventable deaths, and allocating resources to prevent these deaths. The Child Health and Mortality Prevention Surveillance (CHAMPS) network uses a standardized process to define, assign, and code causes of stillbirth and child death (<5 years of age) across the CHAMPS network. A Determination of Cause of Death (DeCoDe) panel composed of experts from a local CHAMPS site analyzes all available individual information, including laboratory, histopathology, abstracted clinical records, and verbal autopsy findings for each case and, if applicable, also for the mother. Using this information, the site panel ascertains the underlying cause (event that precipitated the fatal sequence of events) and other antecedent, immediate, and maternal causes of death in accordance with the *International Classification of Diseases, Tenth Revision* and the World Health Organization death certificate. Development and use of the CHAMPS diagnosis standards—a framework of required evidence to support cause of death determination—assures a homogenized procedure leading to a more consistent interpretation of complex data across the CHAMPS network. This and other standardizations ensures future comparability with other sources of mortality data produced externally to this project. Early lessons learned from implementation of DeCoDe in 5 CHAMPS sites in sub-Saharan Africa and Bangladesh have been incorporated into the DeCoDe process, and the implementation of DeCoDe has the potential to spur health systems improvements and local public health action.

## Teaser

This manuscript details the method for the determination of cause of death in stillbirths and deaths in children under 5 at CHAMPS surveillance sites as well as standardization processes across the network.

Accurate determination of cause of death is the foundation of mortality surveillance. The understanding of how and why children die informs prevention efforts and guides interventions to save lives. Mortality reviews are recognized as important tools in understanding cause of death and prioritizing opportunities for intervention in high-resource countries [1, 2]. Yet, insufficient mortality reviews are conducted in areas where they would be most needed—that is, where the highest child mortality is currently happening [3].

Despite immense strides in the reduction of global under-5 mortality, in 2017 an estimated 5.4 million children died before their fifth birthday and >2.5 million stillbirths occurred. These deaths and stillbirths are disproportionally concentrated in low-resource settings in sub-Saharan Africa and South Asia [4]. Many occur at home without the child ever seeing a healthcare provider. Even when a child is brought to care and subsequently dies in a healthcare setting, cause of death often remains unknown or nonspecific. Clinicians may suspect a condition but may lack the clinical or laboratory resources to confirm their diagnosis with the specificity needed to maximize prevention—for example, the pathogen(s) responsible for pneumonia in a child, or for a maternal chorioamnionitis leading to stillbirth.

Even when the conditions causing death are known, these are often inaccurately or inconsistently recorded on a death certificate. Vital records, including death certificates, exist for less than half of the world’s population [5]. In an assessment of global typology of civil registration and vital statistics, the areas of the world with the highest child mortality often have the poorest performance or no data available [6]. The central aim of the Child Health and Mortality Prevention Surveillance (CHAMPS) network is to fill the gaps in the global understanding of specific causes of under-5 mortality in areas with under-5 mortality >50 per 1000 live births.

Here, we describe the standardized methods, known collectively as the Determination of Cause of Death (DeCoDe) process, used to define, code, and assign causes of under-5 child death and stillbirth across the CHAMPS network.

## OVERVIEW OF DECODE PROCESS

**Figure 1.**
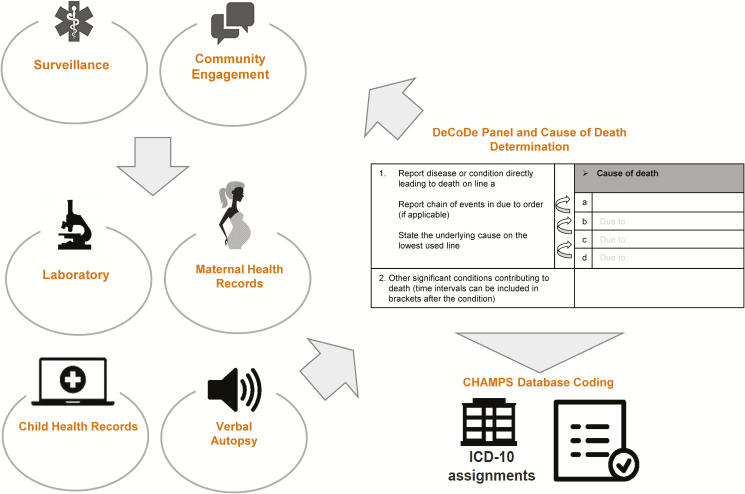
Overview of the determination of cause of death process. Abbreviations: CHAMPS, Child Health and Mortality Prevention Surveillance; DeCoDe, Determination of Causes of Death panel; ICD-10, International Classification of Diseases, Tenth Revision.

Stillbirths and under-5 child deaths from catchment areas in the CHAMPS network that have parental consent (see Salzberg et al in this supplement) undergo postmortem specimen collection through a minimally invasive tissue sampling (MITS) procedure (see Ordi et al in this supplement) to collect tissue for histopathology and detection of pathogens using molecular techniques and immunohistochemistry staining, as well as extensive diagnostic testing. Prior to the MITS procedure, photos and anthropometric measurements are obtained. For each deceased case, CHAMPS sites generate a robust dossier of MITS, histopathology, and laboratory analyses results, along with clinical records (for the deceased child and, if relevant, the mother) and verbal autopsy, to accurately determine cause of death. A panel of experts, known as the DeCoDe panel, comprised by a variety of clinical and laboratorial expertise, thoroughly analyzes and reviews the compiled data in the local context to propose, in light of all available evidence, the most plausible explanation for the underlying cause of death and the fatal sequence of events. This in-depth review is conducted at each CHAMPS surveillance site. Figure 1 outlines the DeCoDe process.

## DEVELOPMENT AND PILOT OF DECODE PROCESS

The CHAMPS DeCoDe panel process was developed as a methodology to review data collected through systematic CHAMPS surveillance activities, elucidate the causal chain of events leading to death, and systematically assign cause of death using standard practices. The DeCoDe process is modeled after death investigation and review panels: There are examples of death investigation and review in high-income settings that use similar approaches [1, 7–9]. Throughout the design and implementation of the DeCoDe process, advisors involved with these reviews were consulted to design an efficient and robust procedure, which could be utilized across all CHAMPS sites.

The DeCoDe panel is composed of clinicians (obstetricians and pediatricians with subspecialty expertise in fields such as neonatology and infectious diseases), microbiologists, pathologists, and medical epidemiologists or other public health specialists. Individual panelists review case information presented in a standardized data packet. After their review, panelists record their cause of death determinations on a standard case report form, in accordance with the World Health Organization (WHO) death certificate, the *International Classification of Diseases, Tenth Revision* (*ICD-10*) [10], and the WHO application of *ICD-10* to deaths during the perinatal period (*ICD-PM*) [11]. A CHAMPS-specific form, modeled from preexisting similar documents, captures the immediate (disease or condition which directly preceded or directly led to death) and the comorbid and underlying (disease or injury that initiated the train of events leading directly to death) causes of death; a series of other useful information is collected such as the duration of each component of the chain of events, the certainty attributed to each diagnosis according to the available results, the utility of every data source for each diagnosis, and the recommendations issued by the panel should they think this death was preventable. For perinatal deaths, this form also includes maternal factors that precipitate or indirectly contribute to the fatal outcome. If panelists submit their individual determinations prior to the panel meeting, a case manager assesses the level of consensus among individual panel members regarding the cause of death determinations. The case manager prioritizes cases with lower concordance for discussion at the full panel meeting to allow for more time for discussion on these cases than on those cases with higher concordance.

During the full panel meeting, a case summary is presented and then the case is opened up for discussion. The panel records the group’s cause of death determination on a standardized group case report form similar to the individual form, and the level of consensus for the determined causes is recorded as unanimous, majority, or no consensus. If consensus is not reached, the case manager determines if any cause of death is recorded or if the case remains undetermined.

Simultaneous with the CHAMPS DeCoDe process development, a pre-CHAMPS MITS pilot study took place in South Africa (see Madhi et al in this supplement). This study investigated deaths in children <12 years old and stillbirths from a health facility and collected similar but slightly more limited data compared to the full CHAMPS protocol. The pre-CHAMPS South African MITS study obtained clinical data (both child and maternal), microbiologic culture and PCR from blood, cerebrospinal fluid, rectal swab, lung, and liver (only culture), and histopathology on brain, liver, and lung; verbal autopsy interviews were not performed. Prior to completion of this study, an external pilot panel through the CHAMPS program office was convened to review a subset of these cases and to test and adapt CHAMPS DeCoDe methodology. Volunteer panel members—pediatricians, pathologists, microbiologists, and epidemiologists—were recruited and trained on death certification and elementary *ICD-10* codes and rules. Panelists reviewed both perinatal and nonperinatal cases and followed the proposed DeCoDe method for 2 panel discussions. In addition, at the end of the study a larger international panel was convened in South Africa to review and adjudicate the cause of deaths for all of the cases enrolled.

The CHAMPS DeCoDe process was refined through lessons learned from this pilot panel experience. Key revisions included making optional the prepanel documentation of individual review results, inclusion of all cases for full panel discussion regardless of individual review concordance, refinement of standard levels of certainty for listing a diagnosis, development of additional guidance to standardize interpretation of data, and identification of additional priority areas for training future CHAMPS panelists, including medical death certification and classification using *ICD-10* and *ICD-PM*.

## DEVELOPMENT OF DIAGNOSIS STANDARDS

Diagnosis standards for DeCoDe were developed to standardize the approach of local panelists in assigning and coding cause of death across diverse CHAMPS surveillance sites and DeCoDe panels. In general, these standards outline how to apply specific CHAMPS clinical and laboratory findings to the diagnoses of conditions contributing to mortality in children and link each diagnosis to its corresponding *ICD-10* code in accordance with international standards for mortality surveillance. In addition, they detail the process of assigning 3 possible levels of certainty to each diagnosis based on the completeness and specificity of available data substantiating the diagnosis. The current CHAMPS Diagnosis Standards (https://champshealth.org/protocols/diagnosis-standards-decode/) include diagnoses that were selected based on several factors including the specific pathogens in the CHAMPS diagnostic array, a list of common causes of neonatal and pediatric death from the Global Burden of Disease Study, and conditions in *ICD-PM* known to cause death in the perinatal period. General standards for level of certainty for diagnosis of a condition were adapted from Population Health Metrics Research Consortium Neonatal and Child Gold Standard Diagnoses (Table 1) [12]. Importantly, the diagnosis standards have been conceived as a “living document,” thus incorporating on an annual basis additional diagnoses based on the lessons learned from their ongoing use.

**Table 1.** General Guidelines for Assigning Level of Certainty for Conditions Affecting Stillbirths, Infants, and Children

Level 1	Level 2	Level 3
Diagnosis of a condition with the highest level of certainty possible for that condition, consisting of (1) highly specific pathological findings or (2) a CHAMPS laboratory test with specific findings and medically observed and documented/clinically observed appropriate illness sign(s).	Diagnosis of a condition with a high level of certainty, consisting of (1) medically observed and documented appropriate illness sign(s) to support the diagnosis or (2) a CHAMPS laboratory test with specific findings and supporting symptoms reported by verbal autopsy.	Conditions that would be considered for diagnosis (ie, from verbal autopsy data alone) but do not meet level 1 or 2 criteria.

Abbreviation: CHAMPS, Child Health and Mortality Prevention Surveillance.

While the guidelines in Table 1 provide a general framework for the consideration of all diagnoses, they have some limitations. The level of certainty for each condition does not indicate causality—a more complicated assessment based on host and other factors—but instead indicates certainty for the diagnosis of a particular condition [13]. The standards cannot elucidate the temporality of events leading to death—the causal chain must be organized on a case-by-case basis in accordance with clinical judgment and *ICD-10* coding standards, and through a consensus-seeking process part of the DeCoDe meetings. In addition, the broadly ranging clinical conditions inherent in CHAMPS cases do not all fit into this framework. For some causes of death (eg, witnessed injuries and poisonings), CHAMPS laboratory tests are not required or available to confirm the diagnosis. Moreover, the significance of many CHAMPS data elements—such as laboratory findings or abstracted clinical symptoms—is subject to interpretation.

Panelists’ ability to apply these standards depends on the nature and completeness of data for any individual case. In some cases, laboratory findings are inconclusive or do not exist so that the only pertinent data derive from clinical record abstractions. In other cases, the highly sensitive laboratory results detect a number of pathogens that may not necessarily be all pathogenic, whereas in other cases the available data are insufficient to assign cause. For some cases, pathological findings are specific for a cause of death that is discordant with an antemortem diagnosis documented in the clinical record. For these types of cases, panelists must consider the entire picture and give more weight to the objective rather than subjective data elements [14]. While these standards do not substitute the clinical and pathological judgment, they do serve as a standard framework for interpreting the case-specific CHAMPS data and offer some objective criteria that inform the inclusion or exclusion of conditions in the causal chain.

**Figure 2.**
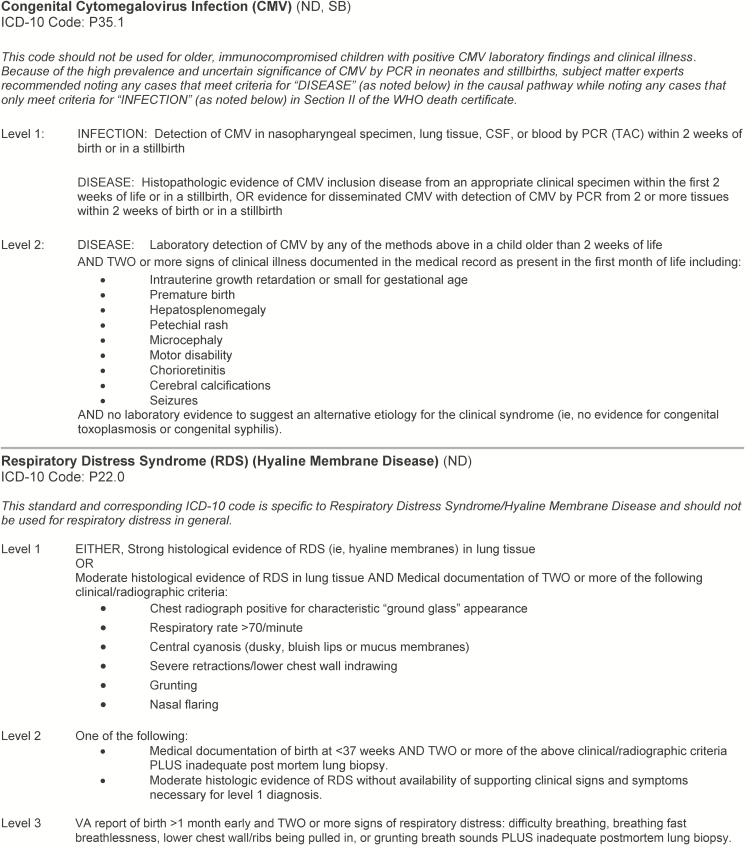
Diagnosis standard examples. The full document can be found https://champshealth.org/protocols/. Neonatal death and stillbirth refer to the type of Child Health and Mortality Prevention Surveillance case for which this standard would be used. Abbreviations: CMV, cytomegalovirus; CSF, cerebrospinal fluid; ICD-10, International Classification of Diseases, Tenth Revision; ND, neonatal death; PCR, polymerase chain reaction; RDS, respiratory distress syndrome; SB, stillbirth; TAC, TaqMan Array Card; VA, verbal autopsy; WHO, World Health Organization.

The CHAMPS Diagnosis Standards for specific conditions (Figure 2) were developed through refinement and application of the general guidelines in Table 1 to specific diagnoses through a multistep process. These included (1) review of existing mortality surveillance or clinical management case definitions from internationally accepted bodies (ie, Centers for Disease Control and Prevention [CDC], Brighton Collaboration Neonatal Infections Working Group, Population Health Metrics Resource Consortium, American College of Obstetrics and Gynecology), as well as individual disease-specific reports; (2) adaptation of these ideal “case definitions” to “diagnosis standards” based on practical consideration of the clinical abstraction, verbal autopsy, and laboratory data available to CHAMPS DeCoDe panels; (3) refinement of each standard based on input from subject matter experts (SMEs) at the CDC, Emory University, and across the CHAMPS network; and (4) further refinement through application to cases from the South Africa pre-CHAMPS pilot with input from additional local site and international SMEs (see Acknowledgments). The diagnosis standards will be updated with additional conditions and revised based on input from local panels and international SMEs on an ongoing basis. While the current diagnosis standards apply to common causes of death for neonates, infants, and children as well as some causes of stillbirth, additional standards are under development to provide more guidance on causes of stillbirth and maternal factors contributing to stillbirth and neonatal mortality.

## DECODE DATA MANAGEMENT, QUALITY CONTROL, AND RESULTS GENERATION

Data collection, management and assimilation into a uniform case packet usable by DeCoDe panelists posed a unique challenge for the CHAMPS network. Information related to each case may include >120 clinical and molecular laboratory results, narrative and discrete pathology diagnoses and findings, and thousands of discrete data fields from clinical record abstractions and verbal autopsy, as well as many documents, photos, and pathology slide images (Table 2). Moreover, housing for primary data must be hosted in countries where CHAMPS operates to minimize privacy and confidentiality concerns for case subjects and families and to comply with host country regulations and laws. Names and other identifying information not essential for execution of the program must be removed from data before submission to the central CHAMPS global data repository.

**Table 2.** Child Health and Mortality Prevention Surveillance Data Types and Sources

Data Type or Source	Specific Content Obtained
Demographic	Age, sex, date of birth, date of death, gestational age, and birth weight
Clinical records	Maternal clinical data including antenatal consultations, delivery, and postpartum information
	Child clinical data including antemortem diagnostics found in medical records and longitudinal information on vaccination and growth history
Verbal autopsy	Caregiver report of signs, symptoms, and narrative of fatal events
MITS procedure	Specimens collected (blood, CSF, NP swab, rectal swab/stool, and tissue biopsies)
	Photographs of face, full body, fingers, and any gross lesions or findings
	Anthropometric measurements: head circumference, length, mid-upper arm circumference
Pathology	Histology of postmortem biopsies (liver, lung, heart, brain, bone marrow)
	Placenta histology (if applicable)
	Immunohistochemistry
	Tissue PCR
Postmortem diagnostics	Microbiology culture (blood, CSF)
	Molecular PCR by TaqMan Array Cards (see Diaz et al in this supplement) (blood, CSF, stool, NP swab, lung tissue)
	HIV PCR testing
	TB PCR testing (lung tissue, stool)
	Malaria testing (thin and thick smears, rapid diagnostic tests)
DeCoDe panel outcomes	Immediate cause of death, comorbid conditions, underlying cause of death; level of certainty, preventability, recommendations for public health action

Abbreviations: CSF, cerebrospinal fluid; DeCoDe, Determination of Causes of Death; HIV, human immunodeficiency virus; MITS, minimally invasive tissue sampling; NP, nasopharyngeal; PCR, polymerase chain reaction; TB, tuberculosis.

Local data management capacity varies across the initial consortium of 5 CHAMPS surveillance sites implementing MITS and DeCoDe. However, all have sufficient capacity to host primary data collection. To reduce DeCoDe data management complexity at sites and to ensure standardization across the CHAMPS network, case management and DeCoDe packet generation are supported via the CHAMPS Data Management Portal (CDMP) web application hosted by the CHAMPS Program Office (PO) at Emory University. The DeCoDe packet is a consolidated document that contains all of the data for each case presented as reports for each data type and source.

Sites collect primary data using tools provided by the CHAMPS Program Office or their own systems for collection. The tools for primary data collection supported include Open Data Kit (Nafundi) for verbal autopsy and the Research Data Capture (REDCap) system (Vanderbilt University) for all other data. Data are extracted from local systems and submitted weekly to the global repository via a CDMP upload page.

As case data accumulate, site and PO staff collaborate on a continuous data quality process that results in high-quality cumulative information being available on each case prior to generation of DeCoDe packets for panel review. Sites and PO staff iteratively track data quality progress throughout the case data curation process with the application of a combination of site- and PO-provided tools, reports, and queries. This process involves data entry validations, data quality scripts/reports that may be implemented by the sites prior to weekly transmissions, automated file validation at the time of submission, and data validation scripts and reporting applied within the PO system. Because this process is applied throughout the active accumulation of data rather than retrospectively at the comprehensive submission of information, it enables higher-quality data within the necessary time targets.

The primary tool for case management and final DeCoDe packet generation is the MITS case management component of the CDMP. This component provides sites and PO staff with a listing of MITS cases that can be filtered and sorted and allows a user to select a specific case to view in detail. Site case managers can manage cases for their site while PO case managers can view and manage cases across the network. The detail view lists a set of reports that users can view or download as a case proceeds through the workup and data become available. Each of the reports is compiled into a DeCoDe packet when case data collection and data quality cycles are complete.

At the time of DeCoDe packet generation, a packet version identifier and associated date/time of creation are recorded in the CDMP database. The packet version identifier is also included on the DeCoDe packet provided to panelists and on final cause of death determinations submitted by the site after the DeCoDe panel has completed its deliberations. The version identifier and associated date enable the generation of a case-level data packet with the most up-to-date information: records dated on or before the packet version date are extracted from the CDMP repository. The packet version identifier format is the concatenation of the case identifier and a 2-part version number (eg, XXAA00001_01_01). The versioning system was designed to support minor and major versions for packets, which occur frequently and rarely, respectively. The minor version supports generating multiple packets with newly updated information *prior to* a DeCoDe panel, whereas the major version links the underlying data and DeCoDe causes of death to a *subsequent* panel or process, which will produce an entirely separate cause of death determination. This latter situation may occur infrequently, for example, when new data become available and reassessing a case is necessary. In addition to the cause of death determinations made by the panel described above collected on a standard DeCoDe results form, the key data elements that were critical in each determination are also recorded. The panel also assesses whether the death in the individual case was preventable and records the public health actions recommended to prevent future deaths. When sites submit DeCoDe results to the CDMP repository, the packet version identifier on each case is matched to the active packet version identifier in the system. If an unknown or inactive packet version identifier is submitted, an error is generated and the PO follows up with the site. By design, the matching of the packet version identifier ensures the DeCoDe determinations submitted were based on the packet of information the panel reviewed. The final curation step for all case data submitted to the CDMP is a review of adherence to *ICD-10* coding methodology and diagnosis standards. The DeCoDe results for each cause determination are reviewed individually to ensure the proper *ICD-10* code was applied. If an incorrect code was assigned, the site is notified of the correct code and requested to change it in the database and resubmit results. Once this review is finished, the case is marked as complete.

## DECODE STANDARDIZATION AND QUALITY ASSURANCE PROCESS

Diagnosis standards and centrally generated data packets are 2 critical aspects of DeCoDe standardization across the CHAMPS network. In addition, the CHAMPS PO provides standard training to all site panelists on the WHO International Form of Medical Certificate of Cause of Death, the classification system, and general usage rules and coding principles following *ICD-10* and *ICD-PM*.

A quality assurance process reviews how DeCoDe panels from each of the diverse CHAMPS settings apply the diagnosis standards to determine cause of death, with the aim of establishing intersite diagnostic reproducibility and concordance across panels. On a regular basis (initially annually) a small subset (1–4) of cases from each study site is selected and shared across the network. Sites review these external cases during a regular panel meeting, and the results are collected centrally and analyzed for concordance across the network. The scoring for the concordance is outlined in Table 3. The concordance results will be used to identify areas needing retraining, revisions to the diagnosis standards, or process method modifications that will improve standardization across the network.

**Table 3.** Consensus Definitions for Child Health and Mortality Prevention Surveillance Quality Assurance Process

Consensus	Definition
Complete consensus	Complete consensus on Part I of the death certificate (all causes in the chain of events leading to death and the sequence of these causes) from all sites/panels
Key element consensus	• Consensus on immediate *and* underlying causes of death with variations only in sequence of events
	• Differences in cause of death assignment at the fourth *ICD-10*–coded digit with the first 3 digits matching
	• Different microbial etiologies selected in an infectious cause of death with >2 contributing microbes selected by the local panel
	• Differences in final selection of specific suspected congenital anomaly if both anomalies are in the same organ system
	• Differences in the use of prematurity and low birth weight codes OR extreme prematurity and extremely low birth weight codes
Partial consensus	Consensus with immediate *or* underlying causes of death
Nonconsensus	No consensus with immediate or underlying causes

Abbreviation: ICD-10, International Classification of Diseases, Tenth Revision.

As a second facet of the quality assurance process, CHAMPS convenes in-person cross-network review of select CHAMPS cases with participants from each of the site panels and invited SMEs. These reviews provide fertile ground for cross-network in-person discussion on challenging aspects of incorporating all sources of evidence, interpreting CHAMPS data, and elucidating the causal chain. These panels will also introduce external SMEs to the DeCoDe process, highlight the importance of local panel insights, and generate discussion around cross-site themes that emerge. Local site teams are then able to compare data-to-action strategies on prevention efforts—from the household level at family follow-up to the community, hospital, and larger spheres.

On an ongoing basis, CHAMPS refines the DeCoDe process and supporting standards to reflect lessons learned through the cross-network case sharing and in-person review processes with the goal of ensuring consistent application of data toward accurate cause of death determination across sites. If procedural changes occur (ie, addition of different types of specimens), methods, standards, and trainings to local sites will be adjusted and updated accordingly.

## DISCUSSION

Currently, most vital registration in low- and middle-income countries (LMICs) has relied on health system records or verbal autopsies. CHAMPS provides a unique opportunity to supplement verbal autopsies with detailed clinical, histological, and microbiological data to more accurately determine cause of death and to validate verbal autopsy algorithms. The challenge is to develop standardized procedures to collate and interpret the data to provide high-quality determinations that mean the same thing across sites and ultimately can form the basis of sustainable and improved cause of death data that can be used by countries for actions to prevent under-5 mortality. The core elements of these standardized procedures in DeCoDe are utilizing *ICD-10* and *ICD-PM* rules and coding, the diagnosis standards, quality data management and control, and the quality assurance procedures.

While there are limitations intrinsic to all death review processes, coordination of DeCoDe processes across the varied CHAMPS sites and within LMIC settings poses some additional challenges. Preparing for and conducting DeCoDe panels is a time- and labor-intensive process both from the programmatic perspective and for panelists. At CHAMPS sites, some local DeCoDe panelists fulfill critical clinical, laboratory, and public health roles with intensive workloads in their respective resource-limited settings. However, as DeCoDe panel discussions focus not just on determination of cause of death but on lessons learned, the process allows participants to address system failures and improve care for future patients—to consider ways to immediately turn data into action and save lives both at facilities and in communities. Panelists derive information from this mortality review process that informs their clinical care and results in the potential for changes to practice—a secondary benefit that is challenging to measure.

Despite a standard approach to the DeCoDe process, there are inherent and preexisting site-to-site differences in data quality and quantity that introduce inconsistencies in the completeness of packet information available to panelists. In addition to the differences in clinical care and diagnostic capabilities between sites, variations in the ways data are collected or recorded in clinical files—including by interviews of clinical staff or family– could lead to the introduction of bias. To help assess these potential sources of bias, the abstraction forms collect information on interviews that occurred and whether the person completing the abstraction was involved in the clinical care of the patient. At the panel meetings, panelists who recall that they were involved in the care of a case disclose this to their colleagues. Given limitations in clinical documentation, the benefit of additional information and insights from these sources outweighs the risk of potential bias.

Elucidating the precise causal pathway and inciting condition(s) or event from single point-in-time results is challenging. While clinical records can be helpful, these records vary widely in their availability, quality, and completeness. Furthermore, patient management and standards of care differ across sites, and the cascade of events leading to death is often intertwined with the local context, care system, and standard of care. This inseparability of cause of death from context underscore the essential role of local perspective in accurate determination of cause of death. However, panelists’ application of local knowledge and insight that is not reflected in data packages can decrease reproducibility of results achieved through the CHAMPS quality assurance process and external review. To address this challenge in reproducibility of results and to capture context-specific knowledge applied by the panel and not captured in data packages, panelists are asked to document any additional information considered on their final cause of death determination form. Local panelists are likely to understand more completely the cases’ clinical and epidemiologic contexts, to have ownership of their local case data and results, and to apply knowledge from the panel process more quickly—a justification for CHAMPS’ centrally supported and standardized but fundamentally site-specific DeCoDe process.

CHAMPS will continue to refine its methods to strengthen quality of conclusions and promote standardization. Nonetheless, the availability of postmortem pathology and laboratory data, added to verbal autopsy and available clinical data, has made it possible to precisely characterize actionable causes [15] and contributors to childhood mortality to a degree that is likely orders of magnitude greater than what was previously available.

## CONCLUSIONS

CHAMPS designed a robust process for determination of cause of death for longitudinal surveillance in multiple sites following principles from other child mortality review processes. These include the use of multidisciplinary expert panel review, standardized systems for data collection, management, curation, and review; development and implementation of specific diagnosis standards; structured approach to panel deliberation with standard forms; and use of already existing international and universally accepted standards for classification and coding of cases. Future analyses of DeCoDe panel outcomes and structured key informant interviews with panelists can reveal the extent to which the process achieves its objective of standardized cause of death determination in addition to fostering discussion on systems deficits and promoting action to reduce child mortality.

## Notes

***Acknowledgments.*** The authors thank the following people for participating on the pilot panels in Atlanta or South Africa and contributing to the development of the diagnosis standards: Lena Camperlengo, Carrie Cottengim, Alexa Erck, Cynthia Whitney, Michele Parsons, Shelley Caltharp, Eleanor Click, Scott Dowell, Christopher Wilson, Jaume Ordi, Sherif Zaki, Martin Hale, Penny Heaton, Eric Houpt, Grace Irimu, Daniel Chandramohan, Nick Kassebum, Keith Klugman, Kim Mulholland, Robert Pattinson, Andi Shane, Peter Swart, Allan Taylor, Kevin Clarke, Maureen Diaz, Jonas Winchell, Robert Pass, Larry Slutsker, Tamara New, Umesh Parashar, Michael Bowen, Jan Vinje, Li DeAlmeida, Eric Mintz, Ciara Sugerman, and Diane Morof. The authors also acknowledge all of the local Determination of Cause of Death panelists for their dedication and commitment and hard work at understanding what is killing children before their fifth birthday and give special thanks to all of the families and communities participating in Child Health and Mortality Prevention Surveillance (CHAMPS).

***Financial support.*** This work was supported by the Bill & Melinda Gates Foundation [OPP1126780].

***Supplement sponsorship.*** This supplement is sponsored by the Emory Global Health Institute and the Bill & Melinda Gates Foundation.

***Potential conflicts of interest.*** S. A. M. has received grants from the Bill & Melinda Gates Foundation, Pfizer, Glaxo Smith Kline, Biovac, Novavax, and Sanofi Pasteur; and honoraria from The Bill & Melinda Gates Foundation. All other authors report no potential conflicts of interest. All authors have submitted the ICMJE Form for Disclosure of Potential Conflicts of Interest. Conflicts that the editors consider relevant to the content of the manuscript have been disclosed.
